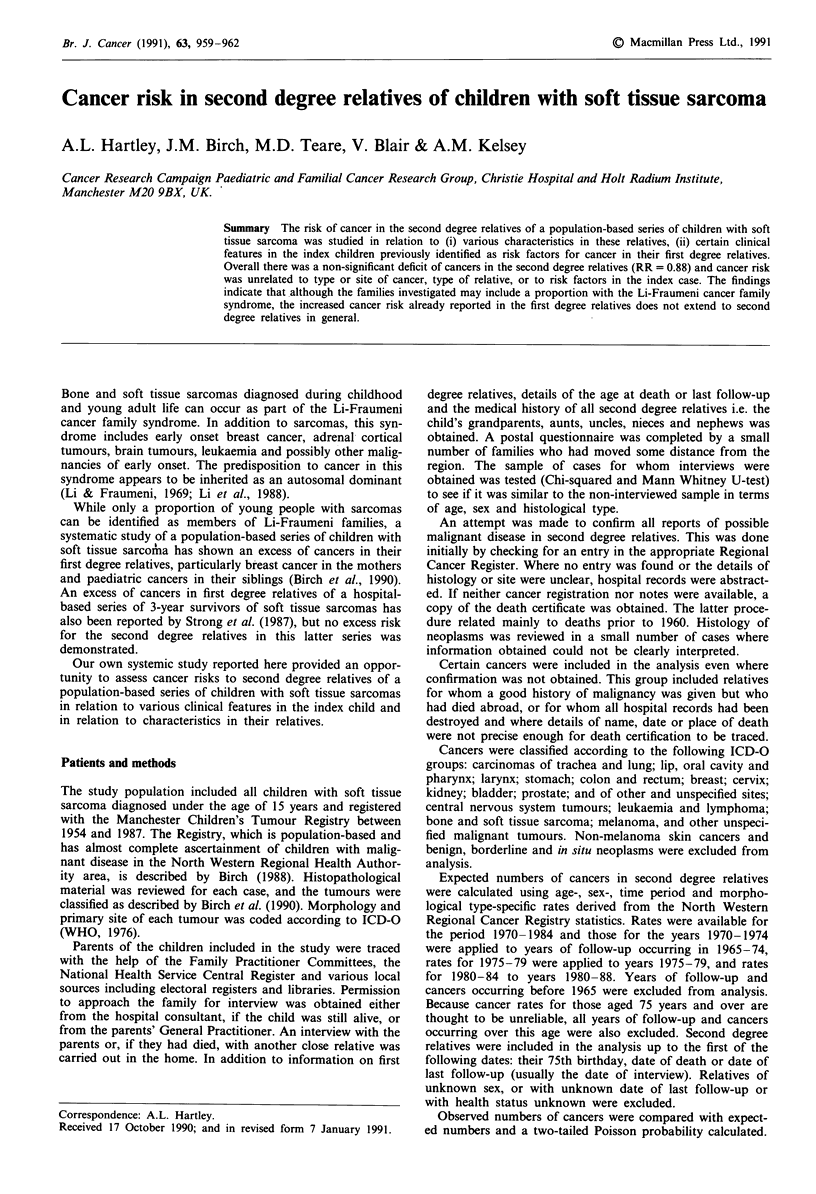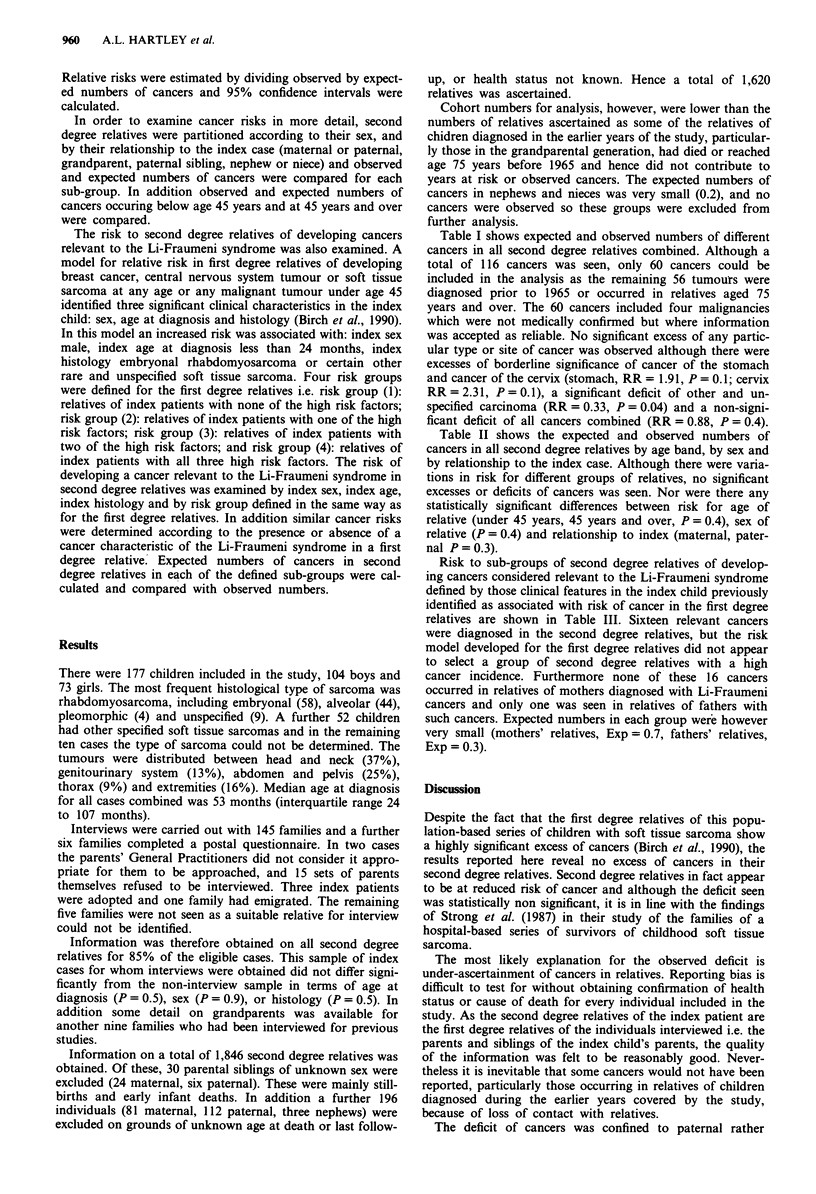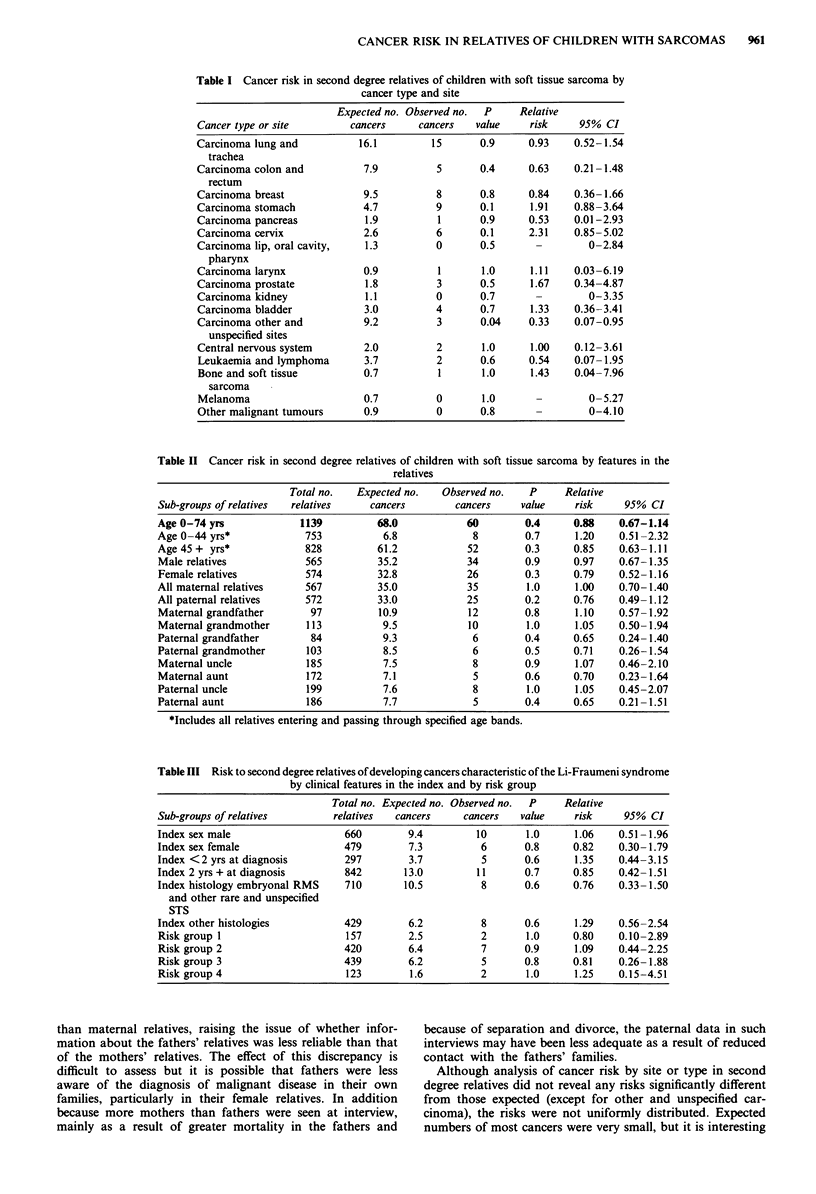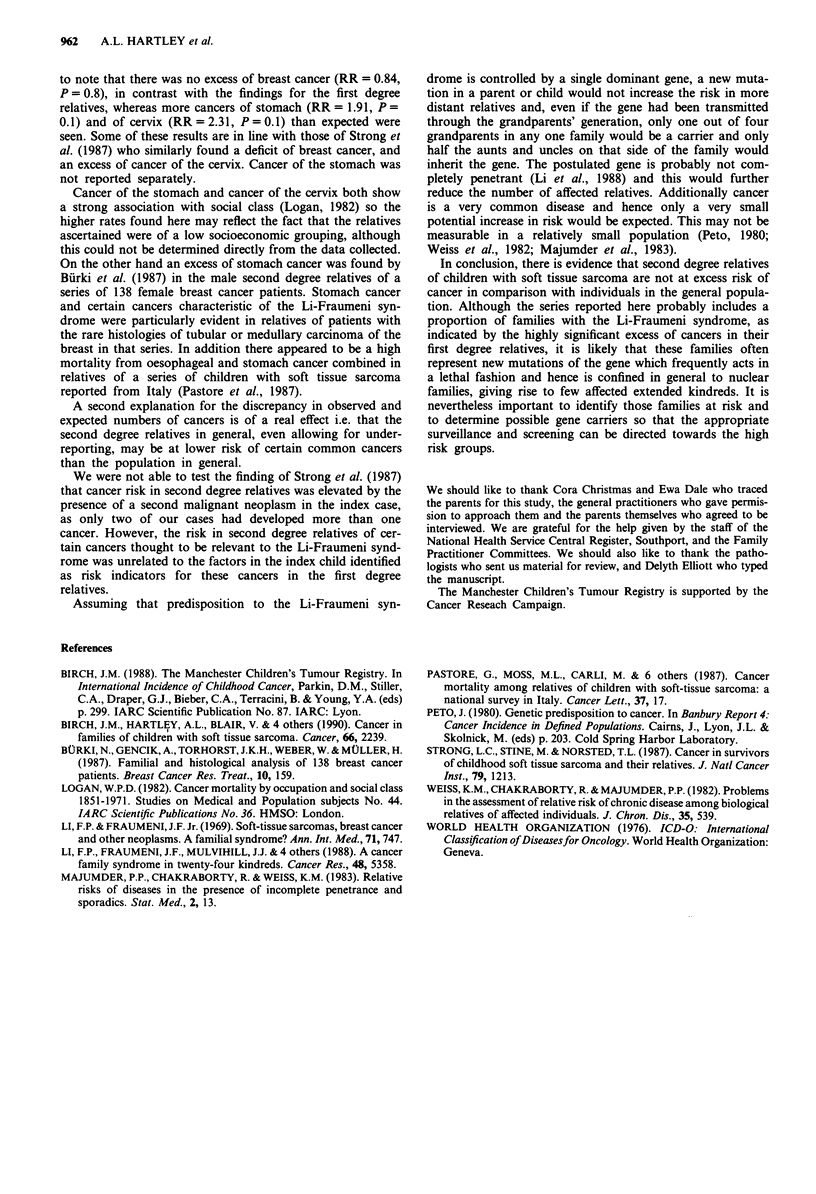# Cancer risk in second degree relatives of children with soft tissue sarcoma.

**DOI:** 10.1038/bjc.1991.209

**Published:** 1991-06

**Authors:** A. L. Hartley, J. M. Birch, M. D. Teare, V. Blair, A. M. Kelsey

**Affiliations:** Cancer Research Campaign Paediatric and Familial Cancer Research Group, Christie Hospital, Manchester, UK.

## Abstract

The risk of cancer in the second degree relatives of a population-based series of children with soft tissue sarcoma was studied in relation to (i) various characteristics in these relatives, (ii) certain clinical features in the index children previously identified as risk factors for cancer in their first degree relatives. Overall there was a non-significant deficit of cancers in the second degree relatives (RR = 0.88) and cancer risk was unrelated to type or site of cancer, type of relative, or to risk factors in the index case. The findings indicate that although the families investigated may include a proportion with the Li-Fraumeni cancer family syndrome, the increased cancer risk already reported in the first degree relatives does not extent to second degree relatives in general.


*Br. J. Cancer* (1991), **63**, 959–962 © Macmillan Press Ltd., 1991

### Cancer risk in second degree relatives of children with soft tissue sarcoma

A.L. Hartley, J.M. Birch, M.D. Teare, V. Blair & A.M. Kelsey

*Cancer Research Campaign Paediatric and Familial Cancer Research Group, Christie Hospital and Holt Radium Institute, Manchester M20 9BX, UK.*

**Summary** The risk of cancer in the second degree relatives of a population-based series of children with soft tissue sarcoma was studied in relation to (i) various characteristics in these relatives, (ii) certain clinical features in the index children previously identified as risk factors for cancer in their first degree relatives. Overall there was a non-significant deficit of cancers in the second degree relatives (RR = 0.88) and cancer risk was unrelated to type or site of cancer, type of relative, or to risk factors in the index case. The findings indicate that although the families investigated may include a proportion with the Li-Fraumeni cancer family syndrome, the increased cancer risk already reported in the first degree relatives does not extend to second degree relatives in general.

Bone and soft tissue sarcomas diagnosed during childhood and young adult life can occur as part of the Li-Fraumeni cancer family syndrome. In addition to sarcomas, this syndrome includes early onset breast cancer, adrenal cortical tumours, brain tumours, leukaemia and possibly other malignancies of early onset. The predisposition to cancer in this syndrome appears to be inherited as an autosomal dominant (Li & Fraumeni, 1969; Li *et al.*, 1988).

While only a proportion of young people with sarcomas can be identified as members of Li-Fraumeni families, a systematic study of a population-based series of children with soft tissue sarcoma has shown an excess of cancers in their first degree relatives, particularly breast cancer in the mothers and paediatric cancers in their siblings (Birch *et al.*, 1990). An excess of cancers in first degree relatives of a hospital-based series of 3-year survivors of soft tissue sarcomas has also been reported by Strong *et al.* (1987), but no excess risk for the second degree relatives in this latter series was demonstrated.

Our own systemic study reported here provided an opportunity to assess cancer risks to second degree relatives of a population-based series of children with soft tissue sarcomas in relation to various clinical features in the index child and in relation to characteristics in their relatives.

### Patients and methods

The study population included all children with soft tissue sarcoma diagnosed under the age of 15 years and registered with the Manchester Children's Tumour Registry between 1954 and 1987. The Registry, which is population-based and has almost complete ascertainment of children with malignant disease in the North Western Regional Health Authority area, is described by Birch (1988). Histopathological material was reviewed for each case, and the tumours were classified as described by Birch *et al.* (1990). Morphology and primary site of each tumour was coded according to ICD-O (WHO, 1976).

Parents of the children included in the study were traced with the help of the Family Practitioner Committees, the National Health Service Central Register and various local sources including electoral registers and libraries. Permission to approach the family for interview was obtained either from the hospital consultant, if the child was still alive, or from the parents' General Practitioner. An interview with the parents or, if they had died, with another close relative was carried out in the home. In addition to information on first degree relatives, details of the age at death or last follow-up and the medical history of all second degree relatives i.e. the child's grandparents, aunts, uncles, nieces and nephews was obtained. A postal questionnaire was completed by a small number of families who had moved some distance from the region. The sample of cases for whom interviews were obtained was tested (Chi-squared and Mann Whitney U-test) to see if it was similar to the non-interviewed sample in terms of age, sex and histological type.

An attempt was made to confirm all reports of possible malignant disease in second degree relatives. This was done initially by checking for an entry in the appropriate Regional Cancer Register. Where no entry was found or the details of histology or site were unclear, hospital records were abstracted. If neither cancer registration nor notes were available, a copy of the death certificate was obtained. The latter procedure related mainly to deaths prior to 1960. Histology of neoplasms was reviewed in a small number of cases where information obtained could not be clearly interpreted.

Certain cancers were included in the analysis even where confirmation was not obtained. This group included relatives for whom a good history of malignancy was given but who had died abroad, or for whom all hospital records had been destroyed and where details of name, date or place of death were not precise enough for death certification to be traced.

Cancers were classified according to the following ICD-O groups: carcinomas of trachea and lung; lip, oral cavity and pharynx; larynx; stomach; colon and rectum; breast; cervix; kidney; bladder; prostate; and of other and unspecified sites; central nervous system tumours; leukaemia and lymphoma; bone and soft tissue sarcoma; melanoma, and other unspecified malignant tumours. Non-melanoma skin cancers and benign, borderline and *in situ* neoplasms were excluded from analysis.

Expected numbers of cancers in second degree relatives were calculated using age-, sex-, time period and morphological type-specific rates derived from the North Western Regional Cancer Registry statistics. Rates were available for the period 1970–1984 and those for the years 1970–1974 were applied to years of follow-up occurring in 1965–74, rates for 1975–79 were applied to years 1975–79, and rates for 1980–84 to years 1980–88. Years of follow-up and cancers occurring before 1965 were excluded from analysis. Because cancer rates for those aged 75 years and over are thought to be unreliable, all years of follow-up and cancers occurring over this age were also excluded. Second degree relatives were included in the analysis up to the first of the following dates: their 75th birthday, date of death or date of last follow-up (usually the date of interview). Relatives of unknown sex, or with unknown date of last follow-up or with health status unknown were excluded.

Observed numbers of cancers were compared with expected numbers and a two-tailed Poisson probability calculated.

Correspondence: A.L. Hartley.
Received 17 October 1990; and in revised form 7 January 1991.

Relative risks were estimated by dividing observed by expected numbers of cancers and 95% confidence intervals were calculated.

In order to examine cancer risks in more detail, second degree relatives were partitioned according to their sex, and by their relationship to the index case (maternal or paternal, grandparent, paternal sibling, nephew or niece) and observed and expected numbers of cancers were compared for each sub-group. In addition observed and expected numbers of cancers occuring below age 45 years and at 45 years and over were compared.

The risk to second degree relatives of developing cancers relevant to the Li-Fraumeni syndrome was also examined. A model for relative risk in first degree relatives of developing breast cancer, central nervous system tumour or soft tissue sarcoma at any age or any malignant tumour under age 45 identified three significant clinical characteristics in the index child: sex, age at diagnosis and histology (Birch *et al.*, 1990). In this model an increased risk was associated with: index sex male, index age at diagnosis less than 24 months, index histology embryonal rhabdomyosarcoma or certain other rare and unspecified soft tissue sarcoma. Four risk groups were defined for the first degree relatives i.e. risk group (1): relatives of index patients with none of the high risk factors; risk group (2): relatives of index patients with one of the high risk factors; risk group (3): relatives of index patients with two of the high risk factors; and risk group (4): relatives of index patients with all three high risk factors. The risk of developing a cancer relevant to the Li-Fraumeni syndrome in second degree relatives was examined by index sex, index age, index histology and by risk group defined in the same way as for the first degree relatives. In addition similar cancer risks were determined according to the presence or absence of a cancer characteristic of the Li-Fraumeni syndrome in a first degree relative. Expected numbers of cancers in second degree relatives in each of the defined sub-groups were calculated and compared with observed numbers.

### Results

There were 177 children included in the study, 104 boys and 73 girls. The most frequent histological type of sarcoma was rhabdomyosarcoma, including embryonal (58), alveolar (44), pleomorphic (4) and unspecified (9). A further 52 children had other specified soft tissue sarcomas and in the remaining ten cases the type of sarcoma could not be determined. The tumours were distributed between head and neck (37%), genitourinary system (13%), abdomen and pelvis (25%), thorax (9%) and extremities (16%). Median age at diagnosis for all cases combined was 53 months (interquartile range 24 to 107 months).

Interviews were carried out with 145 families and a further six families completed a postal questionnaire. In two cases the parents' General Practitioners did not consider it appropriate for them to be approached, and 15 sets of parents themselves refused to be interviewed. Three index patients were adopted and one family had emigrated. The remaining five families were not seen as a suitable relative for interview could not be identified.

Information was therefore obtained on all second degree relatives for 85% of the eligible cases. This sample of index cases for whom interviews were obtained did not differ significantly from the non-interview sample in terms of age at diagnosis ($P = 0.5$), sex ($P = 0.9$), or histology ($P = 0.5$). In addition some detail on grandparents was available for another nine families who had been interviewed for previous studies.

Information on a total of 1,846 second degree relatives was obtained. Of these, 30 parental siblings of unknown sex were excluded (24 maternal, six paternal). These were mainly stillbirths and early infant deaths. In addition a further 196 individuals (81 maternal, 112 paternal, three nephews) were excluded on grounds of unknown age at death or last follow-up, or health status not known. Hence a total of 1,620 relatives was ascertained.

Cohort numbers for analysis, however, were lower than the numbers of relatives ascertained as some of the relatives of chidren diagnosed in the earlier years of the study, particularly those in the grandparental generation, had died or reached age 75 years before 1965 and hence did not contribute to years at risk or observed cancers. The expected numbers of cancers in nephews and nieces was very small (0.2), and no cancers were observed so these groups were excluded from further analysis.

Table I shows expected and observed numbers of different cancers in all second degree relatives combined. Although a total of 116 cancers was seen, only 60 cancers could be included in the analysis as the remaining 56 tumours were diagnosed prior to 1965 or occurred in relatives aged 75 years and over. The 60 cancers included four malignancies which were not medically confirmed but where information was accepted as reliable. No significant excess of any particular type or site of cancer was observed although there were excesses of borderline significance of cancer of the stomach and cancer of the cervix (stomach, $RR = 1.91$, $P = 0.1$; cervix $RR = 2.31$, $P = 0.1$), a significant deficit of other and unspecified carcinoma ($RR = 0.33$, $P = 0.04$) and a non-significant deficit of all cancers combined ($RR = 0.88$, $P = 0.4$).

Table II shows the expected and observed numbers of cancers in all second degree relatives by age band, by sex and by relationship to the index case. Although there were variations in risk for different groups of relatives, no significant excesses or deficits of cancers was seen. Nor were there any statistically significant differences between risk for age of relative (under 45 years, 45 years and over, $P = 0.4$), sex of relative ($P = 0.4$) and relationship to index (maternal, paternal $P = 0.3$).

Risk to sub-groups of second degree relatives of developing cancers considered relevant to the Li-Fraumeni syndrome defined by those clinical features in the index child previously identified as associated with risk of cancer in the first degree relatives are shown in Table III. Sixteen relevant cancers were diagnosed in the second degree relatives, but the risk model developed for the first degree relatives did not appear to select a group of second degree relatives with a high cancer incidence. Furthermore none of these 16 cancers occurred in relatives of mothers diagnosed with Li-Fraumeni cancers and only one was seen in relatives of fathers with such cancers. Expected numbers in each group were however very small (mothers' relatives, $Exp = 0.7$, fathers' relatives, $Exp = 0.3$).

### Discussion

Despite the fact that the first degree relatives of this population-based series of children with soft tissue sarcoma show a highly significant excess of cancers (Birch *et al.*, 1990), the results reported here reveal no excess of cancers in their second degree relatives. Second degree relatives in fact appear to be at reduced risk of cancer and although the deficit seen was statistically non significant, it is in line with the findings of Strong *et al.* (1987) in their study of the families of a hospital-based series of survivors of childhood soft tissue sarcoma.

The most likely explanation for the observed deficit is under-ascertainment of cancers in relatives. Reporting bias is difficult to test for without obtaining confirmation of health status or cause of death for every individual included in the study. As the second degree relatives of the index patient are the first degree relatives of the individuals interviewed i.e. the parents and siblings of the index child's parents, the quality of the information was felt to be reasonably good. Nevertheless it is inevitable that some cancers would not have been reported, particularly those occurring in relatives of children diagnosed during the earlier years covered by the study, because of loss of contact with relatives.

The deficit of cancers was confined to paternal rather

**Table I** Cancer risk in second degree relatives of children with soft tissue sarcoma by cancer type and site

| *Cancer type or site* | *Expected no. cancers* | *Observed no. cancers* | *P value* | *Relative risk* | *95% CI* |
|---|---|---|---|---|---|
| Carcinoma lung and trachea | 16.1 | 15 | 0.9 | 0.93 | 0.52–1.54 |
| Carcinoma colon and rectum | 7.9 | 5 | 0.4 | 0.63 | 0.21–1.48 |
| Carcinoma breast | 9.5 | 8 | 0.8 | 0.84 | 0.36–1.66 |
| Carcinoma stomach | 4.7 | 9 | 0.1 | 1.91 | 0.88–3.64 |
| Carcinoma pancreas | 1.9 | 1 | 0.9 | 0.53 | 0.01–2.93 |
| Carcinoma cervix | 2.6 | 6 | 0.1 | 2.31 | 0.85–5.02 |
| Carcinoma lip, oral cavity, pharynx | 1.3 | 0 | 0.5 | – | 0–2.84 |
| Carcinoma larynx | 0.9 | 1 | 1.0 | 1.11 | 0.03–6.19 |
| Carcinoma prostate | 1.8 | 3 | 0.5 | 1.67 | 0.34–4.87 |
| Carcinoma kidney | 1.1 | 0 | 0.7 | – | 0–3.35 |
| Carcinoma bladder | 3.0 | 4 | 0.7 | 1.33 | 0.36–3.41 |
| Carcinoma other and unspecified sites | 9.2 | 3 | 0.04 | 0.33 | 0.07–0.95 |
| Central nervous system | 2.0 | 2 | 1.0 | 1.00 | 0.12–3.61 |
| Leukaemia and lymphoma | 3.7 | 2 | 0.6 | 0.54 | 0.07–1.95 |
| Bone and soft tissue sarcoma | 0.7 | 1 | 1.0 | 1.43 | 0.04–7.96 |
| Melanoma | 0.7 | 0 | 1.0 | – | 0–5.27 |
| Other malignant tumours | 0.9 | 0 | 0.8 | – | 0–4.10 |

**Table II** Cancer risk in second degree relatives of children with soft tissue sarcoma by features in the relatives

| *Sub-groups of relatives* | *Total no. relatives* | *Expected no. cancers* | *Observed no. cancers* | *P value* | *Relative risk* | *95% CI* |
|---|---|---|---|---|---|---|
| **Age 0–74 yrs** | **1139** | **68.0** | **60** | **0.4** | **0.88** | **0.67–1.14** |
| Age 0–44 yrs* | 753 | 6.8 | 8 | 0.7 | 1.20 | 0.51–2.32 |
| Age 45 + yrs* | 828 | 61.2 | 52 | 0.3 | 0.85 | 0.63–1.11 |
| Male relatives | 565 | 35.2 | 34 | 0.9 | 0.97 | 0.67–1.35 |
| Female relatives | 574 | 32.8 | 26 | 0.3 | 0.79 | 0.52–1.16 |
| All maternal relatives | 567 | 35.0 | 35 | 1.0 | 1.00 | 0.70–1.40 |
| All paternal relatives | 572 | 33.0 | 25 | 0.2 | 0.76 | 0.49–1.12 |
| Maternal grandfather | 97 | 10.9 | 12 | 0.8 | 1.10 | 0.57–1.92 |
| Maternal grandmother | 113 | 9.5 | 10 | 1.0 | 1.05 | 0.50–1.94 |
| Paternal grandfather | 84 | 9.3 | 6 | 0.4 | 0.65 | 0.24–1.40 |
| Paternal grandmother | 103 | 8.5 | 6 | 0.5 | 0.71 | 0.26–1.54 |
| Maternal uncle | 185 | 7.5 | 8 | 0.9 | 1.07 | 0.46–2.10 |
| Maternal aunt | 172 | 7.1 | 5 | 0.6 | 0.70 | 0.23–1.64 |
| Paternal uncle | 199 | 7.6 | 8 | 1.0 | 1.05 | 0.45–2.07 |
| Paternal aunt | 186 | 7.7 | 5 | 0.4 | 0.65 | 0.21–1.51 |

*Includes all relatives entering and passing through specified age bands.

**Table III** Risk to second degree relatives of developing cancers characteristic of the Li-Fraumeni syndrome by clinical features in the index and by risk group

| *Sub-groups of relatives* | *Total no. relatives* | *Expected no. cancers* | *Observed no. cancers* | *P value* | *Relative risk* | *95% CI* |
|---|---|---|---|---|---|---|
| Index sex male | 660 | 9.4 | 10 | 1.0 | 1.06 | 0.51–1.96 |
| Index sex female | 479 | 7.3 | 6 | 0.8 | 0.82 | 0.30–1.79 |
| Index <2 yrs at diagnosis | 297 | 3.7 | 5 | 0.6 | 1.35 | 0.44–3.15 |
| Index 2 yrs + at diagnosis | 842 | 13.0 | 11 | 0.7 | 0.85 | 0.42–1.51 |
| Index histology embryonal RMS and other rare and unspecified STS | 710 | 10.5 | 8 | 0.6 | 0.76 | 0.33–1.50 |
| Index other histologies | 429 | 6.2 | 8 | 0.6 | 1.29 | 0.56–2.54 |
| Risk group 1 | 157 | 2.5 | 2 | 1.0 | 0.80 | 0.10–2.89 |
| Risk group 2 | 420 | 6.4 | 7 | 0.9 | 1.09 | 0.44–2.25 |
| Risk group 3 | 439 | 6.2 | 5 | 0.8 | 0.81 | 0.26–1.88 |
| Risk group 4 | 123 | 1.6 | 2 | 1.0 | 1.25 | 0.15–4.51 |

than maternal relatives, raising the issue of whether information about the fathers' relatives was less reliable than that of the mothers' relatives. The effect of this discrepancy is difficult to assess but it is possible that fathers were less aware of the diagnosis of malignant disease in their own families, particularly in their female relatives. In addition because more mothers than fathers were seen at interview, mainly as a result of greater mortality in the fathers and because of separation and divorce, the paternal data in such interviews may have been less adequate as a result of reduced contact with the fathers' families.

Although analysis of cancer risk by site or type in second degree relatives did not reveal any risks significantly different from those expected (except for other and unspecified carcinoma), the risks were not uniformly distributed. Expected numbers of most cancers were very small, but it is interesting to note that there was no excess of breast cancer (RR = 0.84, $P = 0.8$), in contrast with the findings for the first degree relatives, whereas more cancers of stomach (RR = 1.91, $P = 0.1$) and of cervix (RR = 2.31, $P = 0.1$) than expected were seen. Some of these results are in line with those of Strong *et al.* (1987) who similarly found a deficit of breast cancer, and an excess of cancer of the cervix. Cancer of the stomach was not reported separately.

Cancer of the stomach and cancer of the cervix both show a strong association with social class (Logan, 1982) so the higher rates found here may reflect the fact that the relatives ascertained were of a low socioeconomic grouping, although this could not be determined directly from the data collected. On the other hand an excess of stomach cancer was found by Bürki *et al.* (1987) in the male second degree relatives of a series of 138 female breast cancer patients. Stomach cancer and certain cancers characteristic of the Li-Fraumeni syndrome were particularly evident in relatives of patients with the rare histologies of tubular or medullary carcinoma of the breast in that series. In addition there appeared to be a high mortality from oesophageal and stomach cancer combined in relatives of a series of children with soft tissue sarcoma reported from Italy (Pastore *et al.*, 1987).

A second explanation for the discrepancy in observed and expected numbers of cancers is of a real effect i.e. that the second degree relatives in general, even allowing for under-reporting, may be at lower risk of certain common cancers than the population in general.

We were not able to test the finding of Strong *et al.* (1987) that cancer risk in second degree relatives was elevated by the presence of a second malignant neoplasm in the index case, as only two of our cases had developed more than one cancer. However, the risk in second degree relatives of certain cancers thought to be relevant to the Li-Fraumeni syndrome was unrelated to the factors in the index child identified as risk indicators for these cancers in the first degree relatives.

Assuming that predisposition to the Li-Fraumeni syndrome is controlled by a single dominant gene, a new mutation in a parent or child would not increase the risk in more distant relatives and, even if the gene had been transmitted through the grandparents' generation, only one out of four grandparents in any one family would be a carrier and only half the aunts and uncles on that side of the family would inherit the gene. The postulated gene is probably not completely penetrant (Li *et al.*, 1988) and this would further reduce the number of affected relatives. Additionally cancer is a very common disease and hence only a very small potential increase in risk would be expected. This may not be measurable in a relatively small population (Peto, 1980; Weiss *et al.*, 1982; Majumder *et al.*, 1983).

In conclusion, there is evidence that second degree relatives of children with soft tissue sarcoma are not at excess risk of cancer in comparison with individuals in the general population. Although the series reported here probably includes a proportion of families with the Li-Fraumeni syndrome, as indicated by the highly significant excess of cancers in their first degree relatives, it is likely that these families often represent new mutations of the gene which frequently acts in a lethal fashion and hence is confined in general to nuclear families, giving rise to few affected extended kindreds. It is nevertheless important to identify those families at risk and to determine possible gene carriers so that the appropriate surveillance and screening can be directed towards the high risk groups.

We should like to thank Cora Christmas and Ewa Dale who traced the parents for this study, the general practitioners who gave permission to approach them and the parents themselves who agreed to be interviewed. We are grateful for the help given by the staff of the National Health Service Central Register, Southport, and the Family Practitioner Committees. We should also like to thank the pathologists who sent us material for review, and Delyth Elliott who typed the manuscript.

The Manchester Children's Tumour Registry is supported by the Cancer Reseach Campaign.

### References

BIRCH, J.M. (1988). The Manchester Children's Tumour Registry. In *International Incidence of Childhood Cancer*, Parkin, D.M., Stiller, C.A., Draper, G.J., Bieber, C.A., Terracini, B. & Young, Y.A. (eds) p. 299. IARC Scientific Publication No. 87. IARC: Lyon.

BIRCH, J.M., HARTLEY, A.L., BLAIR, V. & 4 others (1990). Cancer in families of children with soft tissue sarcoma. *Cancer*, **66**, 2239.

BÜRKI, N., GENCIK, A., TORHORST, J.K.H., WEBER, W. & MÜLLER, H. (1987). Familial and histological analysis of 138 breast cancer patients. *Breast Cancer Res. Treat.*, **10**, 159.

LOGAN, W.P.D. (1982). Cancer mortality by occupation and social class 1851-1971. Studies on Medical and Population subjects No. 44. *IARC Scientific Publications No. 36.* HMSO: London.

LI, F.P. & FRAUMENI, J.F. Jr. (1969). Soft-tissue sarcomas, breast cancer and other neoplasms. A familial syndrome? *Ann. Int. Med.*, **71**, 747.

LI, F.P., FRAUMENI, J.F., MULVIHILL, J.J. & 4 others (1988). A cancer family syndrome in twenty-four kindreds. *Cancer Res.*, **48**, 5358.

MAJUMDER, P.P., CHAKRABORTY, R. & WEISS, K.M. (1983). Relative risks of diseases in the presence of incomplete penetrance and sporadics. *Stat. Med.*, **2**, 13.

PASTORE, G., MOSS, M.L., CARLI, M. & 6 others (1987). Cancer mortality among relatives of children with soft-tissue sarcoma: a national survey in Italy. *Cancer Lett.*, **37**, 17.

PETO, J. (1980). Genetic predisposition to cancer. In *Banbury Report 4: Cancer Incidence in Defined Populations*. Cairns, J., Lyon, J.L. & Skolnick, M. (eds) p. 203. Cold Spring Harbor Laboratory.

STRONG, L.C., STINE, M. & NORSTED, T.L. (1987). Cancer in survivors of childhood soft tissue sarcoma and their relatives. *J. Natl Cancer Inst.*, **79**, 1213.

WEISS, K.M., CHAKRABORTY, R. & MAJUMDER, P.P. (1982). Problems in the assessment of relative risk of chronic disease among biological relatives of affected individuals. *J. Chron. Dis.*, **35**, 539.

WORLD HEALTH ORGANIZATION (1976). *ICD-O: International Classification of Diseases for Oncology*. World Health Organization: Geneva.